# High-risk children and social isolation: the importance of family functioning

**DOI:** 10.3389/fpsyg.2023.1190438

**Published:** 2023-06-22

**Authors:** Maria Fernanda Vieira, Maria Dalva Barbosa Baker Méio, Ana Beatriz Rodrigues Reis, Letícia Duarte Villela, Maura Calixto Cecherelli de Rodrigues, Fátima Cristiane Pinho de Almeida Di Maio Ferreira, Letícia Baptista de Paula Barros, Roozeméria Pereira Costa, Elaine Rego Menezes, Camila Oliveira Campos, Maria Elisabeth Lopes Moreira, Saint Clair S. Gomes-Junior

**Affiliations:** ^1^Follow-Up Clinic, Department of Neonatology, Instituto Nacional da Saúde da Mulher, da Criança e do Adolescente Fernandes Figueira, Fundação Oswaldo Cruz (FIOCRUZ), Rio de Janeiro, Brazil; ^2^Pesquisa Clínica Aplicada, Instituto Nacional da Saúde da Mulher, da Criança e do Adolescente Fernandes Figueira, Fundação Oswaldo Cruz (FIOCRUZ), Rio de Janeiro, Brazil; ^3^High-risk Newborn Follow-Up Clinic, Department of Pediatrics, Hospital Universitário Pedro Ernesto, Faculdade de Ciências Médicas, Universidade do Estado do Rio de Janeiro, Rio de Janeiro, Brazil; ^4^Follow-Up Clinic, Hospital Gaffrée Guinle, Universidade Federal do Estado do Rio de Janeiro (UNIRIO), Rio de Janeiro, Brazil; ^5^Statistics Division, Instituto Nacional da Saúde da Mulher, da Criança e do Adolescente Fernandes Figueira, Fundação Oswaldo Cruz (FIOCRUZ), Rio de Janeiro, Brazil

**Keywords:** child behavior, premature infant, neurodevelopmental disorders, COVID-19, social isolation, parenting, family

## Abstract

High-risk newborns are exposed to neonatal conditions such as prematurity, very low birth weight, and congenital malformations that can affect development and behavior. Coronavirus disease 2019 (COVID-19) restraint and control measures have been identified as important stressor events and cumulative risk factors for behavioral changes in these children. This study examined social isolation-related factors that contribute to internalizing and externalizing behavior problems in children already at risk for neurodevelopmental disorders. This cross-sectional, multicenter study included 113 children (18 months to 9 years) who were followed in reference services for neonatal follow-up in tertiary units of the public health system in the city of Rio de Janeiro, Brazil. Behavior was assessed using the child behavior checklist, and a structured questionnaire was used to assess sociodemographic aspects. In the bivariate analysis, prematurity was associated with externalizing problems and change in eating habits with internalizing problems. The logistic model indicated that both parents having completed high school and both sharing care of the child were protective factors for behavioral problems; however, reports of sleep problems and living with another child were risk factors. In conclusion, the study identified internalizing and externalizing behavior problems related to prematurity and aspects of family structure and routine in children at risk. The findings confirm the importance of family functioning for child health and family-centered interventions.

## 1. Introduction

Early exposure to stressful events has been associated with an increased risk of developing behavioral changes (Kushner et al., 2016). The coronavirus disease 2019 (COVID-19) pandemic was an early adverse experience for many children, resulting in disruption of routines, confinement at home, separation from friends, and feelings of fear in the home environment (Panda et al., 2020; Urban et al., 2022). Studies have reported a high prevalence of depression, anxiety, irritability, boredom, attention problems, fear, and sleep disturbances among children during this pandemic (Ma et al., 2021; Panda et al., 2020).

Many studies have described the emotional health of specific population subgroups, such as those with autism spectrum disorder, attention-deficit hyperactivity disorder, cancer and other neurodiverse conditions, or chronic physical health conditions (Samji et al., 2022). We found few studies (Ehrler et al., 2021; Monnier et al., 2021; Bailhache et al., 2022) on the effects of isolation on high-risk children. These high-risk newborns are exposed to neonatal conditions that require treatment in a neonatal intensive care unit (NICU); although necessary for life-sustaining care, the NICU environment involves repeated exposures to painful, invasive procedures and multiple handling (Anand and Scalzo, 2000). The main criteria for identifying high-risk are prematurity <32 weeks gestational age, very low birth weight, neonatal encephalopathy, meningitis, and malformations that affect neurodevelopment (Doyle et al., 2014). Owing to the higher risk of adverse developmental and behavioral outcomes, these children should be followed in specialized outpatient clinics after hospital discharge to allow early identification of changes and intervention (Doyle et al., 2014).

In addition to the aforementioned biological risks, social issues such as low socioeconomic status and low maternal education are also risk factors for neurodevelopment. According to the cumulative risk model, when an individual is exposed to more than one risk factor, these factors have complex interacting effects (Sun et al., 2021). The greater the number of risk factors, the greater the prevalence of clinical behavior problems (Appleyard et al., 2005). Studies of changes in child behaviors due to the COVID-19 pandemic have focused on countries in Europe, Asia, and North America (Samji et al., 2022), and a few studies of high-risk children (Ehrler et al., 2021; Monnier et al., 2021; Bailhache et al., 2022) have been conducted in developed countries. Therefore, the effects observed in these studies may not reflect the reality of children living in underdeveloped or developing countries. Regarding education, a UNICEF survey showed that Latin American and Caribbean countries were most affected between March 2020 and February 2021. During this period, Brazil ranked fifth among countries with the most days of school closure because of the pandemic, behind Panama, El Salvador, Bangladesh, and Bolivia (UNICEF, 2021). Schools are considered important not only for learning but also for the health, safety, and well-being of children (UNICEF, 2021).

Identifying factors associated with behavioral problems during the COVID-19 pandemic may facilitate the design of mental health programs in critical situations. Therefore, this study aimed to analyze factors contributing to behavioral problems in children already at risk for neurodevelopmental disorders, considering biological, sociodemographic, and sociofamilial routine aspects of social isolation.

## 2. Materials and methods

### 2.1. Study design, population, and place

This multicenter cross-sectional study was conducted in three tertiary hospitals of the public health system in the city of Rio de Janeiro, Brazil, including children at risk admitted to the NICU of these hospitals and followed up in the respective outpatient clinics. The study was conducted between September and December 2020.

### 2.2. Eligibility criteria and sample size

The sample was composed of all children between 18 months and 12 years of age who were followed up in these units and who attended the consultations when they resumed regular care during the study period, following the recommended contingency measures. The exclusion criteria were genetic syndromes and cerebral palsy with greater impairment [levels III, IV, and V of the gross motor function classification system (GMFCS)] (Palisano et al., 1997).

### 2.3. Variables

The following blocks of variables were considered:

1. Demographic and perinatal aspects: sex, age at assessment, gestational age, birth weight, bronchopulmonary dysplasia, intracranial hemorrhage, retinopathy of prematurity, perinatal asphyxia, and necrotizing enterocolitis.
2. Sociodemographic and economic aspects: parents’ schooling, the presence of the father living with the child, main caregiver of the child, living with another child, employment status of the parents, unemployment of the mother or father because of the pandemic, government assistance (Bolsa Família or Auxílio Emergencial), COVID-19 diagnosis in the family nucleus, and attendance at daycare or school before the pandemic was declared. Bolsa Família is a government cash transfer program for poor and extremely poor families in Brazil (Lucas et al., 2022), and Auxílio Emergencial was a socioeconomic program created to mitigate the effects of the COVID-19 outbreak in Brazil (Albani et al., 2022).
3. Child’s routine: **caregiver’s** perception of worsening sleep quality and change in eating habits. Exclusively for preschool and school children, the following were also included: receiving educational activities from daycare/school to be done at home, online classes (synchronous or asynchronous), maintaining communication with friends, and leisure activities at home such as using electronic games, listening to music and dancing, and playing games with the family (such as board games).
4. Behavioral assessment: child behavior checklist [CBCL] syndromic categories.

### 2.4. Data collection

Information on birth and perinatal morbidity was obtained from the medical records. A structured questionnaire was used with items about social, family, and routine aspects of the child.

To assess the child’s primary caregiver, the respondent was asked to indicate whether it was the mother, father, both (mother and father), or someone else. It was explained that the person responsible for the child’s care, such as feeding, education, guidance on hygiene habits, behavior, and social interaction, is considered a caregiver.

To assess changes in eating habits, the respondent should consider changes in schedules, increased consumption of snacks or processed foods, and skipping one or more meals during the pandemic.

To assess children’s behavior, we used the CBCL instrument (Achenbach, 1991), which was translated and validated for the Brazilian population (Bordin et al., 1995). The CBCL covers the age from 18 months to 18 years and is divided into two forms: one for children aged 18 months to 5 years (CBCL-1), with 100 items, and another for those ranged 6–18 years (CBCL-2), with 113 items. The protocols are completed by the child’s parents or guardian.

The CBCL scores generate syndrome scales, which refer to a set of items that tend to occur together. In CBCL-1, four syndromes are characterized as internalizing problems (i.e., emotionally reactive, anxious/depressed, somatic complaints, and withdrawn), two as externalizing problems (i.e., attention problems and aggressive behavior), sleep problems, and stress. The CBCL-2 scores generate three syndrome scales to characterize internalizing problems (anxious/depressed, withdrawn/depressed, and somatic complaints) and two for externalizing problems (rule-breaking behavior and aggressive behavior), in addition to social problems, thought problems, attention problems, and stress. Regardless of which CBCL model is being scored, the total behavior problem score is the sum of all items on the instrument. The score is categorized as normal, borderline, or clinical. For the syndrome scales, scores of 65–69 points are considered borderline, and > 70 points are considered clinically significant. For the internalizing, externalizing, and total problem scales, scores of 60–63 points are considered borderline, and ≥ 64 points are clinically significant. For analysis, participants were grouped into clinically/borderline (>60 points) and normal according to their CBCL scale scores.

### 2.5. Statistical analysis

The database was prepared using the EPIINFO program version 7.2.4.0, and statistical analyses were performed using IBM SPSS Statistics version 23 (IBM Corp., Armonk, NY, United States). The children were divided into three age groups: toddlers (up to 35 months), preschoolers (36 months to 71 months), to whom the CBCL-1 was administered, and schoolchildren (≥6 years), to whom the CBCL-2 was administered. Data from toddlers were analyzed separately from preschoolers and schoolchildren because of expected differences in feeding, play, and socialization routines. Categorical variables were described by frequency of occurrence and numerical variables by median and interquartile range. Pearson’s chi-squared test, with Fisher’s correction when appropriate, was used to compare demographic, perinatal, sociofamilial assessment, and child routine variables among behavior categories found on the CBCL, considering a significance level of <0.05. A multivariate logistic model was used to evaluate the association between the independent variables of demographics, perinatal, sociofamilial, and child routine changes with the dependent variables of the internalizing scale and the externalizing scale. Both regression models included age and all variables with a value of p of <0.20 in the bivariate analyses. Borderline and clinical scores on the CBCL were combined into a single dependent variable. A significance level of <0.05 was considered in the final model.

### 2.6. Ethical issues

This study was approved by the ethics committees of the participating institutions (Registration Nos. 35934820.3.0000.5269, 35934820.3.3002.5259, and 35934820.3.3001.5258), and children were included in the study after their legal guardians signed the informed consent form.

## 3. Results

During the study period, 133 children were eligible, of whom six were excluded (genetic syndromes, n = 3; cerebral palsy levels III–V of the GMFCS, n = 3). The parents of the three children refused to participate, and 11 children did not complete the tests. Thus, 113 children were evaluated and distributed as follows: 48 (42.5%) up to 3 years of age, 31 (27.4%) preschoolers, and 34 (30.1%) schoolchildren. The mean age of the participating children was 4 years, ranging from 18 months to 9 years. **Most questionnaires were answered by the mothers (90.3%), while another 6 (5.3%) by the fathers and 5 (4.4%) by the grandmothers.**

In the sample, the children were born preterm (74.3%), were small for gestational age (SGA, 31%), and had bronchopulmonary dysplasia (15%), intracranial hemorrhage (12.4%), necrotizing enterocolitis (6.2%), perinatal asphyxia (8.8%), and other associated comorbidities (23%). Regarding the mothers’ education, 17.7% had completed elementary school, 9.7% had incomplete high school, 61.1% had completed high school, and 11.5% had a college degree. As for the fathers, 21.2% had completed elementary school, 17.7% had incomplete high school, 47.8% had completed high school, and 6.2% had a college degree. Regarding financial support from the government, 29.2% reported receiving funds from Bolsa Família, **a Brazilian regular assistance**, and 55.8% had access to Auxílio Emergencial, **an emergency government assistance due to the pandemic.** Regarding COVID, 24.8% of the families reported that a family member living in the same house had been affected. In this sample, 18.8% of toddlers, 90.3% of preschoolers, and all schoolchildren attended daycare or school before the pandemic. Parents reported that 31.9% of children’s sleep quality was affected by the pandemic, and 42.5% had a change in eating habits.

Toddlers and preschoolers have a comparable distribution of the proportions of normal, borderline, and clinical scores on the internalizing scale, with 20.8% of toddlers and 22.6% of preschoolers scoring in the clinical range. A high percentage of school-aged children (44.1%) scored in the same range. On the externalizing scale, a higher proportion was noted in the clinical range in toddlers (31.3%) compared with preschoolers (9.7%) and schoolchildren (20.6%) (Table 1).

**Table 1:** Distribution of CBCL-1 and CBCL-2 components according to the classification in the syndrome scales.

	Normal	Borderline	Clinical
	*n* (%)	*n* (%)	*n* (%)
CBCL-1 – Toddler (< 3 years)			
*Internalizing Scale*	28 (58.3)	10 (20.8)	10 (20.8)
Emotionally reactivity	33 (68.8)	6 (12.5)	9 (18.8)
Anxious / Depressed	38 (79.2)	7 (14.6)	3 (6.3)
Somatic complaints	45 (93.8)	1 (2.1)	2 (4.2)
Withdrawn	34 (70.8)	8 (16.7)	6 (12.5)
*Externalizing Scale*	23 (47.9)	10 (20.8)	15 (31.3)
Attention problems	28 (58.3)	7 (14.6)	13 (27.1)
Aggressive behavior	37 (77.1)	8 (16.7)	3 (6.3)
Sleep problems	39 (81.3)	2 (4.2)	7 (14.6)
Stress	37 (77.1)	2 (4.2)	9 (18.8)
*Total Problems*	23 (47.9)	8 (16.7)	17 (35.4)
CBCL-1 – Preschoolers (3 to 6 years)			
*Internalizing Scale*	19 (61.3)	5 (16.1)	7 (22.6)
Emotionally reactivity	26 (83.9)	2 (6.5)	3 (9.7)
Anxious / Depressed	21 (67.7)	6 (19.4)	4 (12.9)
Somatic complaints	26 (83.9)	5 (16.1)	0
Withdrawn	24 (77.4)	3 (9.7)	4 (12.9)
*Externalizing Scale*	21 (67.7)	7 (22.6)	3 (9.7)
Attention problems	22 (71.0)	6 (19.4)	3 (9.7)
Aggressive behavior	28 (90.3)	1 (3.2)	2 (6.5)
Sleep problems	27 (87.1)	3 (9.7)	1 (3.2)
Stress	25 (80.6)	3 (9.7)	3 (9.7)
*Total Problems*	21 (67.7)	2 (6.5)	8 (25.8)
CBCL-2 – Schoolchildren (≥ 6 years)			
*Internalizing Scale:*	14 (41.2)	5 (14.7)	15 (44.1)
Anxious / Depressed	22 (64.7)	6 (17.6)	6 (17.6)
Withdrawn / Depressed	27 (79.4)	5 (14.7)	2 (5.9)
Somatic complaints	25 (73.5)	6 (17.6)	3 (8.8)
*Externalizing Scale*	21 (61.8)	6 (17.6)	7 (20.6)
Rule-breaking behavior	33 (97.1)	0	1 (2.9)
Aggressive behavior	24 (70.6)	7 (20.6)	3 (8.8)
Social problems	30 (88.2)	3 (8.8)	1 (2.9)
Problems of thought	28 (82.4)	2 (5.9)	4 (11.8)
Attention problems	27 (79.4)	4 (11.8)	3 (8.8)
Stress	23 (67.6)	4 (11.8)	7 (20.6)
*Total Problems*	15 (44.1)	10 (29.4)	9 (26.5)

On the internalizing scale, a higher prevalence in the clinical range for toddlers was found for emotional reactivity (18.8%) and for preschoolers for anxiety/depression and withdrawal/isolation (both at 12.9%). Among schoolchildren, 35% scored outside the normal range for the anxiety/depression component (17.6% for the clinical range). On the externalizing scale, the most common component in the clinical range was attention problems for toddlers (27.1%) and preschoolers (9.7%), and it was aggressive behavior for schoolchildren (8.8%) (Table 1).

The variable relating to maternal schooling was significant in both the internalizing and externalizing scales for toddlers. In the externalizing scale in the same age group, prematurity was also significant (Table 2).

**Table 2:** Distribution of perinatal conditions, sociodemographic, and routine aspects according to the internalizing and externalizing scales of CBCL for toddlers.

Variables	Toddlers						
	*n* = 48 *n* (%)	Internalizing			Externalizing		
		Normal^**1**^ *n* = 28 *n* (%)	Altered^**1**^ *n* = 20 *n* (%)	Value of *p*	Normal^1^ *n* = 23 *n* (%)	Altered^1^ *n* = 25 *n* (%)	Value of *p*
Perinatal and neonatal conditions							
Preterm	29 (60.4)	16 (57.1)	13 (65.0)	0.58	10 (43.5)	19 (76.0)	0.02*
Small for gestational age	14 (29.2)	8 (28.6)	6 (30.0)	0.91	5 (21.7)	9 (36.0)	0.27
Comorbidities^2^	15 (31.3)	7 (25.0)	8 (40.0)	0.26	9 (39.1)	6 (24.0)	0.25
Sociodemographic aspects							
Male gender	23 (47.9)	14 (50.0)	9 (45.0)	0.73	11 (47.8)	12 (48.0)	0.99
Mother completed high school	32 (66.7)	23 (82.1)	9 (45.0)	0.007*	20 (87.0)	12 (48.0)	0.006*†
Father completed high school	23 (53.5)	15 (57.7)	8 (47.1)	0.49	13 (56.5)	10 (50.0)	0.66
Mother and father concluded high school	19 (39.6)	13 (46.4)	6 (30.0)	0.25	10 (43.5)	9 (36.0)	0.59
Presence of the father living with the family	44 (91.7)	26 (92.9)	18 (90.0)	0.72	22 (95.7)	22 (88.0)	0.33
Mother and father shared childcare	6 (12.5)	3 (10.7)	3 (15.0)	0.68†	5 (21.7)	1 (4.0)	0.09†
Lives with another child	28 (58.3)	15 (53.6)	13 (65.0)	0.42	11 (47.8)	17 (68.0)	0.15
Covid-19 in the family	13 (27.1)	9 (32.1)	4 (20.0)	0.51†	7 (30.4)	6 (24.0)	0.61
Enrollment in daycare/school: Yes	9 (18.8)	4 (14.3)	5 (25.0)	0.46†	4 (17.4)	5 (20.0)	1.00†
Economic aspects							
Mother works	21 (43.8)	12 (42.9)	9 (45.0)	0.88	9 (39.1)	12 (48.0)	0.53
Father works	40 (87.0)	24 (88.9)	16 (84.2)	0.64	20 (87.0)	20 (87.0)	1.00
Unemployment due to pandemic	5 (10.4)	2 (7.1)	3 (15.0)	0.63†	2 (8.7)	3 (12.0)	1.00†
Received Bolsa-Família (Family Grant)	12 (25.0)	7 (25.0)	5 (25.0)	1.00	4 (17.4)	8 (32.0)	0.32†
Received Auxílio Emergencial (Emergency Assistance)	24 (50.0)	12 (42.9)	12 (60.0)	0.24	11 (47.8)	13 (52.0)	0.77
Routine							
Sleep problems	13 (27.1)	5 (17.9)	8 (40.0)	0.08	5 (21.7)	8 (32.0)	0.42
Change in eating habits	14 (29.2)	8 (28.6)	6 (30.0)	0.91	7 (30.4)	7 (28.0)	0.85

*p < 0.05; †Fisher exact test; ^1^Normal – classified as within the expected range; ^1^Altered – classified as borderline or clinic; ^2^Comorbidities: congenital dacryocystocele, malformations (gastroschisis, esophagus atresia, heart disease), cerebral palsy (2 cases of GMFCS level I) and congenital infections (exposure to syphilis and 8 with exposure to chikungunya virus).

In preschoolers and schoolchildren, paternal schooling was related to the internalizing and externalizing scales. Changes in eating habits were associated with internalizing problems (Table 3).

**Table 3:** Distribution of perinatal conditions, sociodemographic and routine aspects according to the internalizing and externalizing scales of CBCL for preschoolers and schoolchildren.

Variables	Preschoolers and schoolchildren						
	*n* = 65 *n* (%)	Internalizing			Externalizing		
		Normal^1^ *n* = 33 *n* (%)	Altered^1^ *n* = 32 *n* (%)	Value of *p*	Normal^1^ *n* = 42 n (%)	Altered^1^ *n* = 23 *n* (%)	Value of *p*
Perinatal and neonatal conditions							
Preterm	55 (84.6)	28 (84.8)	27 (84.4)	0.95	35 (83.3)	20 (87.0)	0.69
Small for gestational age	21 (32.3)	11 (33.3)	10 (31.3)	0.85	17 (40.5)	4 (17.4)	0.09†
Comorbidities^2^	11 (16.9)	6 (18.2)	5 (15.6)	0.78	7 (16.7)	4 (17.4)	1.00†
Sociodemographic aspects							
Male gender	32 (49.2)	17 (51.5)	15 (46.9)	0.70	22 (52.4)	10 (43.5)	0.49
Mother completed high school	50 (76.9)	25 (75.8)	25 (78.1)	0.82	31 (73.8)	19 (82.6)	0.54†
Father completed high school	38 (61.3)	24 (75.0)	14 (46.7)	0.02*	31 (79.5)	7 (30.4)	< 0.005*
Mother and father concluded high school	31 (47.7)	20 (60.6)	11 (34.4)	0.03*	25 (59.5)	6 (26.1)	0.01*
Presence of the father living with in the family	54 (83.1)	27 (81.8)	27 (84.4)	0.78	34 (81.0)	20 (87.0)	0.53
Mother and father caregivers (both)	22 (33.8)	14 (42.4)	8 (25.0)	0.13	18 (42.9)	4 (17.4)	0.05†
Lives with another child	34 (52.3)	16 (48.5)	18 (56.3)	0.53	20 (47.6)	14 (60.9)	0.30
Covid-19 in the family	15 (23.1)	8 (24.2)	7 (21.9)	0.82	10 (23.8)	5 (21.7)	0.85
Enrollment in daycare/school: Yes	62 (95.4)	31 (93.9)	31 (96.9)	0.57	40 (95.2)	22 (95.7)	0.93
Economic aspects							
Mother works	40 (61.5)	21 (63.6)	19 (59.4)	0.72	24 (57.1)	16 (69.6)	0.32
Father works	47 (78.3)	23 (76.7)	24 (80.0)	0.75	29 (78.4)	18 (78.3)	0.99
Unemployment due to pandemic	11 (16.9)	4 (12.1)	7 (21.9)	0.33†	6 (14.3)	5 (21.7)	0.44
Receives Bolsa-Família (Family Grant)	21 (32.3)	7 (21.2)	14 (43.8)	0.05	13 (31.0)	8 (34.8)	0.75
Receives Auxílio Emergencial (Emergency Assistance)	39 (60.0)	19 (57.6)	20 (62.5)	0.68	24 (57.1)	15 (65.2)	0.52
Routine							
Sleep problems	23 (35.4)	8 (24.2)	15 (46.9)	0.05	14 (33.3)	9 (39.1)	0.64
Change in eating habits	34 (52.3)	12 (36.4)	22 (68.8)	0.009*	19 (45.2)	15 (65.2)	0.12
School tasks at home	53 (81.5)	28 (84.8)	25 (78.1)	0.48	37 (88.1)	16 (69.6)	0.06
Online classes	23 (35.4)	10 (30.3)	13 (40.6)	0.38	15 (35.7)	8 (34.8)	0.94
Communicated with friends	34 (52.3)	19 (57.6)	15 (46.9)	0.38	24 (57.1)	10 (43.5)	0.29
Distraction							
Use of electronic games	50 (76.9)	25 (75.8)	25 (78.1)	0.82	33 (78.6)	17 (73.9)	0.67
Listen to music, dance	35 (53.8)	20 (60.6)	15 (46.9)	0.26	22 (52.4)	13 (56.5)	0.74
Games with the family	21 (32.3)	12 (36.4)	9 (28.1)	0.47	16 (38.1)	5 (21.7)	0.17

*p < 0.05; †Fisher exact test; ^1^Normal – classified as within the expected range; ^1^Altered – classified as borderline or clinic; ^2^Comorbidities: congenital cataracts, malformations (diaphragmatic hernia, hypoplasia of the abdominal muscles, pulmonary hypoplasia, cystic adenomatous malformation, 2 with heart disease), cerebral palsy (2 cases of GMFCS level II) and congenital infections (exposure to toxoplasmosis and exposure to syphilis).

The logistic model indicated that having both parents who completed high school was associated with a lower chance of clinical or borderline problems on the internalizing scale of the CBCL; however, the presence of sleep problems was associated with a higher chance of such problems. Children whose parents took care of the children together were less likely to report clinical or borderline problems on the externalizing scale of the CBCL; however, living with another child contributed to a greater chance of externalizing problems (Table 4).

**Table 4:** Initial and final logistic regression model of the relation between the perinatal, socio-demographic, economic and child’s routine variables with the CBCL classification (normal or altered) as dependent variable for the Internalizing and Externalizing Scales.

Variables of the logistic model	OR (CI95%)	Value of *p*
Internalizing scale		
Initial model		
Age	1.012 (0.997–1.027)	0.130
Mother and father concluded high school	0.465 (0.203–1.065)	0.070
Mother and father shared childcare	0.629 (0.224–1.762)	0.378
Family Grant (Bolsa-Família)	1.495 (0.600–3.726)	0.388
Sleep problems	2.382 (0.954–5.943)	0.063
Changes in eating habits	1.450 (0.599–3.510)	0.410
Final model		
Mother and father concluded high school	0.404 (0.183–0.894)	0.025
Sleep problems	2.982 (1.283–6.931)	0.011
Externalizing scale		
Initial model		
Age	1.016 (0.991–1.042)	0.204
Preterm	2.050 (0.744–5.647)	0.165
Small for gestational age	0.636 (0.247–1.639)	0.349
Mother and father concluded high school	0.532 (0.216–1.306)	0.168
Mother and father shared childcare	0.236 (0.069–0.804)	0.021
Lives with another child	1.934 (0.764–4.894)	0.164
Changes in eating habits	1.134 (0.449–2.868)	0.790
School tasks at home	0.275 (0.065–1.173)	0.081
Games with the family	0.389 (0.134–1.128)	0.082
Final model		
Mother and father concluded high school	0.464 (0.202–1.066)	0.070
Mother and father shared childcare	0.179 (0.059–0.546)	0.003
Lives with another child	2.394 (1.030–5.567)	0.043

## 4. Discussion

This study analyzed factors that contribute to behavioral problems in at risk children, taking into account biological, sociodemographic, and routine-related aspects in social isolation. In the bivariate analysis, prematurity was associated with externalizing behaviors in toddlers, and changes in eating habits were associated with internalizing problems in preschoolers and schoolchildren. The model indicated that having both parents who completed high school and both sharing childcare were protective factors for behavioral problems; however, living with another child and reporting sleep problems were risk factors.

The association between prematurity and externalizing behavior in toddlers was attributed to the high prevalence of attention problems, which comprise the externalizing scale. The findings are consistent with studies conducted before the pandemic. Cosentino-Rocha et al. (2014) assessed infants between 18 and 36 months of age and found an effect of prematurity on attention problems. The authors emphasized the importance of early identification of such problems to promote interventions (Cosentino-Rocha et al., 2014). Attention deficits are one of the most common neurobehavioral problems after premature birth, may persist into adulthood, and be associated with negative emotional and educational outcomes (Retzler et al., 2019).

In this study, frequencies in the clinical range for externalizing problems were higher in toddlers and internalizing problems in schoolchildren. Giannotti et al. (2022) also found a higher prevalence of externalizing problems in younger children during the pandemic. According to Basten et al. (2016), younger children have limited communication skills and may use externalizing behaviors, such as aggressiveness and disruptive behaviors, to express their feelings. They added that the development of cognitive skills and the resulting ability to regulate emotions tended to reduce aggressiveness and increase internalizing problems in older children (Fanti and Henrich, 2010).

Social isolation limited families’ access to support networks and allowed for a broader observation of family functioning. In the present study, results showed that children whose parents shared childcare had fewer externalizing problems, confirming the importance of the presence of support in the family nucleus. The division of child-related care is one of the components of coparenting, defined as the joint and reciprocal involvement of both parents in the education, and decisions about their children’s lives (Feinberg, 2003; He et al., 2021; Lucassen et al., 2021). Giannotti et al. (2022) found an indirect relationship between reciprocal parental support and children’s externalizing behavior during the pandemic, and this relationship was mediated by stress. Their results showed that parental stress was an important predictor of children’s externalizing problems, and that stress was reduced with coparenting. The association between externalizing problems and parental stress had been reported before the pandemic (Jones et al., 2017). During social isolation, coparenting has been identified by other authors as an important protective factor in mitigating parents’ mental health problems (Li et al., 2022), contributing to their better sense of well-being and greater ability to provide emotional support to their children (Urban et al., 2022). Programs aimed at strengthening effective family support networks and building parents’ emotional skills would be one way to help parents cope with stressful situations (Li et al., 2022; Urban et al., 2022), with positive effects on their children’s behavior.

In this study, an association was found between parents’ education (maternal and paternal) and children’s behavior. Higher education is likely to enhance various skills, including problem-solving and coping skills (Hosokawa and Katsura, 2017), which may have been crucial for parents to face the challenges of the pandemic. According to Li et al. (2022), parents with lower educational levels had more daily problems and mental health issues with sudden changes because of social isolation. In addition, families with higher education levels tend to seek more knowledge about child development and invest in both educational materials and communication time with their children, which affect language (Zhang, 2012; Hosokawa and Katsura, 2017).

Contrary to our expectations, the results indicated a greater chance of externalizing problems for those living with another child. These findings corroborate reports from studies of family dynamics during the pandemic. According to Sama et al. (2021), parents reported more sibling fights during social isolation and associated fights with reduced home space and lower maternal education (Sama et al., 2021). During the pandemic, children of families with more than three children had more mental health problems (Luijten et al., 2021), and parents with more than one child had more daily hassles and stress symptoms (Li et al., 2022).

Changes in family routines affected behaviors. Children with sleep problems were twice as likely to have internalizing problems, confirming other findings that sleep quality affected children’s psychological well-being during the pandemic (Ng and Ng, 2022). Sleep disturbances are of concern because they are widely observed in the major forms of childhood psychopathology, including anxiety, depression, and attention disorders; sleep and these disorders have complex associations (Gregory and Sadeh, 2012). Changes in eating habits have also been found to be associated with internalizing problems in preschool and school children. One of the main causes of unhealthy lifestyles is the lack of a structured daily routine (Ng and Ng, 2022); thus, parents should establish and maintain a healthy routine that includes sleep and mealtimes (Segre et al., 2021).

This study has some limitations. First, it has a cross-sectional design; therefore, it lacks information on the pre-pandemic behavior of these children. Second, appointments were resumed with a limit on the number of children treated per shift, which reduced the number of participants. Third, families more affected by the pandemic may not have had the health or financial conditions to attend the hospital, as well as it is possible that parents concerned about their children’s behavior might have sought care more often, which may have led to a selection bias, but not a systematic one.

Despite these limitations, this study stands out because it adds to our knowledge of the behavior of children considered at high-risk in the context of a developing country, where many accumulate biological risk and social vulnerability. In addition, the study was conducted in reference hospitals for neonatal and follow-up care, with trained teams and in full compliance with the measures recommended by health authorities during the pandemic. The face-to-face evaluation allowed the inclusion of families with limited access to the Internet or electronic devices who are often not represented in surveys that use online questionnaires.

In conclusion, the results found have implications for professionals who care for these children because the identification of factors allows for preventive intervention and early guidance of families. The study identified internalizing and externalizing behavior problems in at risk children and that these were related to prematurity, family structure, and routine. Risk factors identified included parental educational disadvantage, number of children in the home, and lack of a structured daily routine, whereas the presence of reciprocal parental caregiving support was a protective factor. The findings confirm the importance of understanding family structure to design policies and interventions that reduce negative influences on behavior and promote positive family experiences.

## Data availability statement

The raw data supporting the conclusions of this article will be made available by the authors, without undue reservation.

## Ethics statement

The studies involving human participants were reviewed and approved by (1) Instituto Fernandes Figueira - Fundação Oswaldo Cruz, (2) Hospital Universitário Gaffrée e Guinle - UNIRIO and (3) Hospital Universitário Pedro Ernesto - Universidade do Estado do Rio de Janeiro. The patients/participants provided their written informed consent to participate in this study.

## Author contributions

MMé and MV contributed with the conception of the study. MMé was responsible for the coordination of the study, contributing with the analysis and interpretation of the data, the writing of the manuscript, and its critical revision. MV, AR, LV, MR, FA, RC, EM, and CC contributed with material preparation and data collection. AR, RC, EM, and CC applied the CBCL. LP and SG-J performed the statistical analysis and contributed with the interpretation of the results. MV wrote the first draft of the manuscript. AR and MMé contributed with the subsequent revisions. LV, MR, FA, RC, EM, CC, and MMo contributed with the critical revision of the manuscript. All authors contributed to the article and approved the submitted version.

## Funding

This work was supported by Programa INOVA FIOCRUZ (grant no. VPPCB-005-FIO-20-2-67 / 48400640976574), and by FAPERJ (project no. 210.925/2021).

## Conflict of interest

The authors declare that the research was conducted in the absence of any commercial or financial relationships that could be construed as a potential conflict of interest.

## Publisher’s note

All claims expressed in this article are solely those of the authors and do not necessarily represent those of their affiliated organizations, or those of the publisher, the editors and the reviewers. Any product that may be evaluated in this article, or claim that may be made by its manufacturer, is not guaranteed or endorsed by the publisher.
